# Association of common mental disorders and related multimorbidity with subsequent labor market marginalization among refugee and Swedish-born young adults

**DOI:** 10.3389/fpubh.2023.1054261

**Published:** 2023-03-16

**Authors:** Jiaying Chen, Ellenor Mittendorfer-Rutz, Lisa Berg, Marie Nørredam, Marit Sijbrandij, Peter Klimek

**Affiliations:** ^1^Section for Science of Complex Systems, CeDAS, Medical University of Vienna, Vienna, Austria; ^2^Complexity Science Hub Vienna, Vienna, Austria; ^3^Division of Insurance Medicine, Department of Clinical Neuroscience, Karolinska Institutet, Stockholm, Sweden; ^4^Department of Public Health Sciences, Stockholm University, Stockholm, Sweden; ^5^Centre for Health Equity Studies, Karolinska Institutet, Stockholm University, Stockholm, Sweden; ^6^Danish Research Centre for Migration, Ethnicity, and Health (MESU), Section for Health Services Research, Department of Public Health, University of Copenhagen, Copenhagen, Denmark; ^7^Section of Immigrant Medicine, Department of Infectious Diseases, University Hospital Hvidovre, Copenhagen, Denmark; ^8^Department of Clinical, Neuro-and Developmental Psychology, WHO Collaborating Centre for Research and Dissemination of Psychological Interventions, Amsterdam Public Health Research Institute, Vrije Universiteit, Amsterdam, Netherlands

**Keywords:** common mental disorders, disability pension, unemployment, refugee, disease network, multimorbidity

## Abstract

**Background:**

Common mental disorders (CMDs), multimorbidity, and refugee status are associated with poor labor market outcome. Little is known about how these factors interact in young adults.

**Objective:**

We aimed to i) investigate whether the association of CMDs and multimorbidity with labor market marginalization (LMM) differs between refugee and Swedish-born young adults and ii) identify diagnostic groups with particularly high risk for LMM.

**Methods:**

This longitudinal registry-based study included individuals aged 20–25 years followed from 2012 to 2016 in Sweden (41,516 refugees and 207,729 age and sex-matched Swedish-born individuals). LMM was defined as granted disability pension (DP) or > 180 days of unemployment (UE). A disease co-occurrence network was constructed for all diagnostic groups from 2009 to 2011 to derive a personalized multimorbidity score for LMM. Multivariate logistic regression was used to estimate odds ratios of LMM in refugee and Swedish-born youth as a function of their multimorbidity score. The relative risk (RR, 95% CI) of LMM for refugees with CMDs compared to Swedish-born with CMDs was computed in each diagnostic group.

**Results:**

In total, 5.5% of refugees and 7.2% of Swedish-born with CMDs were granted DP; 22.2 and 9.4%, respectively received UE benefit during follow-up. While both CMDs and multimorbidity independently elevated the risk of DP considerably in Swedish-born, CMDs but not multimorbidity elevated the risk of UE. Regarding UE in refugees, multimorbidity with the presence of CMDs showed stronger estimates. Multimorbidity interacted with refugee status toward UE (*p* < 0.0001) and with CMDs toward DP (*p* = 0.0049). Two diagnostic groups that demonstrated particularly high RR of UE were schizophrenia, schizotypal and delusional disorders (RR [95% CI]: 3.46 [1.77, 6.75]), and behavioral syndromes (RR [95% CI]: 3.41 [1.90, 6.10]).

**Conclusion:**

To combat LMM, public health measures and intervention strategies need to be tailored to young adults based on their CMDs, multimorbidity, and refugee status.

## Introduction

Young adults in Europe are at increased risk of experiencing labor market marginalization (LMM) (1, 2). While there are various definitions of LMM, here we adopt the definition of long-term unemployment (UE) and disability pension (DP) (3). Health-related aspects can be better captured by this definition as the decision on granting a DP requires medical assessments. Within young individuals with LMM, substantial disparity between migrants, particularly refugees and native-born have been reported in numerous studies (4–7). Exposure to trauma and adversities, uncertainties of settlement and post-migration living difficulties make young refugees particularly vulnerable and more susceptible to adverse health outcomes and poor social integration when compared to their native-born peers (4, 8, 9). As in many European countries, and also worldwide, the population of refugees increased in recent decades (8, 10), understanding the relation between health status and LMM among young refugees is of critical importance. This may help to implement strategies to promote inclusion and participation of refugee youth in the labor market, reduce social disadvantage within refugee populations and potentially improve economic stability of these countries.

Common mental disorders (CMDs), i.e., depression, anxiety, and stress-related disorders (11), are a leading cause of disability in young adults. These mental disorders contribute considerably to functional health loss in all ages worldwide and correlate with increased risk of LMM next to the need of welfare support in later life (11–13). CMDs are particularly prevalent in refugees, which may be related to trauma exposure, post-migration conditions, and social exclusion (14). The negative impact of the migration experience are long-lasting (15). Low socioeconomic status and educational attainment heighten difficulties of labor market engagement among young refugees with CMDs (3, 4, 8). Prior studies revealed a higher risk of LMM among young refugees than native-born with CMDs within a large-scale registry-based cohort in Sweden (4, 5).

Previous studies reported that CMDs were associated with increased multimorbidity, defined as the coexistence of two or more conditions in an individual within a given timespan (16–20). The increased burden of multimorbidity has become a public concern, in particular among individuals with lower socioeconomic status or social deprivation (21). Many studies on assessing multimorbidity relied on questionnaires and focused on older adult populations (22). Despite the risk of multimorbidity being highly correlated with age, multimorbidity also has a significant impact on the social development of young adults and risk of LMM (16, 22). While both CMDs and multimorbidity have been found to elevate the risk of LMM in youths (3, 13, 22–24). There is a social gradient in the association of CMDs with higher morbidities (16). Yet, whether the relationship between comorbid mental/somatic disorders and LMM differs by refugee status has received little interest in the literature to date, let alone how these factors interact with each other.

Instruments used to evaluate disease co-occurrences are heterogeneous and often measured by counting the total number of pre-determined chronic conditions (18, 25). In this study, we utilized a disease network approach to quantify multimorbidity in individuals. Disease networks consist of nodes that represent individual diseases and links indicating a statistical tendency for two diseases to co-occur in patients (26). A strength of the network approach is that all diagnoses across the entire spectrum are accounted for within a single framework without having to make assumptions about possible underlying common causes. We hypothesized that CMDs and multimorbidity, conceptualized by indicators derived from disease networks, would correlate with LMM differently in refugees and Swedish-born youths. A better understanding of these concerted interactions might inform about effective social and community support to prevent or reduce LMM in refugees, and policies facilitating access to work for refugees. In the current study, we aimed i) to evaluate the association of multimorbidity, CMD, and refugee status with subsequent LMM and ii) to identify diagnostic groups with an elevated risk of LMM in refugee and Swedish-born young adults with CMDs.

## Methods

A longitudinal cohort study was carried out using information from combined Swedish registers. A total of 256,326 young adults aged between 20 and 25 years at baseline (December 31, 2011) and living in Sweden from January 01, 2009 to December 31, 2011, were identified in the Total Population Register. Refugees were 1:5 matched with Swedish-born based on age, sex, and area of residence (i.e., big cities, medium cities, or rural area). Refugees were identified based on reasons for settlement in Sweden. These individuals received a residence permit in Sweden due to one of the following reasons: refugee status as defined by the Geneva Convention of Refugees, on “humanitarian grounds,” “in need of protection,” or “family reunification” (27). The matched comparison group consisted of Swedish-born counterparts with both parents being native-born Swedes. We excluded individuals with ongoing DP at baseline (*n* = 5,658) and individuals who were not living in Sweden at baseline (*n* = 1,423). In the end, the study population consisted of a total of 249,245 young adults with 41,516 refugees and 207,729 Swedish-born counterparts. To evaluate the association of CMDs, multimorbidity, and LMM risk, these 249,245 individuals were followed in terms of DP and UE from January 01, 2012 until December 31, 2016. The diagnostic criteria and the eligibility of DP remained the same throughout the study period from January 01, 2009 to December 31, 2016. Ethical approval for this study was obtained from the Regional Ethical Review Board, Karolinska Institutet, Stockholm (nr 2007/762-31).

## Data sources

The Longitudinal Integration Database for Health Insurance and Labor Market Studies (LISA) contains information regarding sociodemographic, employment, and social benefits since 1990. Migration and refugee status, including dates and reasons for migrations, were obtained from the longitudinal database for integration studies (STATIV). The National Patient Register (NPR) contains the primary and secondary diagnostic information in both inpatient and specialized outpatient care settings since 1987 and 2001, respectively. Disease prevalence was defined according to the International Classification of Diseases Tenth Edition (ICD-10) in the NPR registers. Mortality data was collected from the Cause of Death Register. Date and duration of sickness absences and DP were collected from Microdata for Analysis of Social Security (MIDAS), with the earliest available DP information since 1994. Prescribed medication information was obtained from the Prescribed Drug Register. The quality of the register data has been validated by prior studies (28).

### Measure of common mental disorders (CMDs)

CMDs were classified by the International Classification of Diseases version 10 (ICD-10) codes F32–F33 and F40–F43, indicating depressive episodes, recurrent depressive disorder, phobic anxiety disorder, other anxiety disorder, obsessive-compulsive disorder, reaction to severe stress, and adjustment disorders, from inpatient or specialized outpatient records, or any prescribed antidepressant medication according to the Anatomical Therapeutic Chemical (ATC) Classification (ATC code N06A) from 2009 to 2011 (3).

### Assessment of multimorbidity

A phenotypic disease network approach to obtain a multimorbidity score was performed based on disease prevalence between January 01, 2009 and December 31, 2011 in the NPR. Primary and secondary diagnoses were classified by 114 ICD-10 diagnostic groups as defined by the WHO; see Supplementary Table 1 (29). For each pair of diagnostic groups, their tendency to co-occur was statistically assessed (30).

### Measure of labor market marginalization (LMM)

The measure of LMM was based on information on granted DP (regardless of grade) and UE collected during the follow-up period from January 01, 2012 to December 31, 2016. UE was recorded and administered by the Swedish Public Employment Service and registered in LISA. Cases of UE were defined as individuals who were registered as unemployed for more than 180 days in a calendar year.

### Covariates

Demographic information on age (continuous), sex (male/female), family situation (categorical), education level (categorical), and area of residence (categorical) were collected at baseline. Family situation was categorized as: married or cohabitant without children, married or cohabitant with children, single without children living at home, single with children living at home, and youth younger than the age of 20 years living at home. Education levels were classified by “0–9 years,” “10–12 years,” “> 12 years” or unknown. Area of residence indicated whether individuals resided in big cities, medium-sized cities, or rural areas. Big cities referred to Stockholm, Gothenburg, and Malmö; medium-sized cities were defined as cities with more than 90,000 residents within 30 kilometers from the city center; rural area indicated all the remaining cities or villages in Sweden. History of spells of sickness absence of > 90 net days and UE during January 01, 2009 and December 31, 2011 were obtained from the registers described above.

### Statistical analysis and multimorbidity score

A multimorbidity network analysis of the entire cohort based on ICD-10 diagnostic groups observed between January 01, 2009 and December 31, 2011 was performed to derive personal multimorbidity scores. The constructed multimorbidity network consisted of nodes and links, indicating ICD-10 diagnostic groups (nodes) and their statistical tendency to co-occur with other diseases as measured by the logarithmic odds ratio for each pair of diagnostic groups (link strength), respectively. Only diagnostic groups that have been diagnosed in at least 100 individuals and pairs of diagnostic groups that have occurred at least 40 times were kept for the network. Disparity filter, an algorithm for filtering non-significant associations by node-specific threshold values, was applied to address multiple testing and to mitigate the influence of statistical biases regarding the link strengths. A detailed description of the method has been previously given (31).

The multimorbidity score was computed from the network for each individual as the weighted average of the LMM risk (measured at baseline) in the disease network neighborhood of that individual; see Supplementary Methods and Supplementary Figure 1. The set of diagnoses that are statistically significant of at least one of the given individual's diagnoses at baseline is referred to as the network neighborhood. The LMM risk in a given diagnostic group was computed as the frequency of LMM in all patients with this diagnosis at baseline. The multimorbidity score was computed as the weighted sum of these LMM risks with weights proportional to the link strength.

Multivariate logistic regression was used to evaluate the association of multimorbidity score and LMM in refugees with or without CMDs and Swedish-born with or without CMDs. In addition to the matching variables (age, sex, and area of residence), education, history of sickness absence, and history of UE were included in the multivariate logistic regression. The multimorbidity score was classified into three categories: “zero,” “low,” and “high,” according to the median of the non-zero scores. Individuals in the “zero” category were either a) healthy individuals with no prevalent diagnosis in the specialized health care system or b) individuals with rare diseases or diagnoses that had no significant association with other diagnostic groups in the network. A multimorbidity score below or above the median of all non-zero scores corresponded to categories “low” and “high,” respectively. A composite variable was created by combining the information for refugee and CMD status across strata for multimorbidity scores as the exposure of the multivariate logistic regression. Swedish-born without CMDs in the multimorbidity category “zero” of the multimorbidity score was defined as the reference group. The interaction of refugee status, CMDs, and multimorbidity score was evaluated by ANOVA.

**Figure 1:**
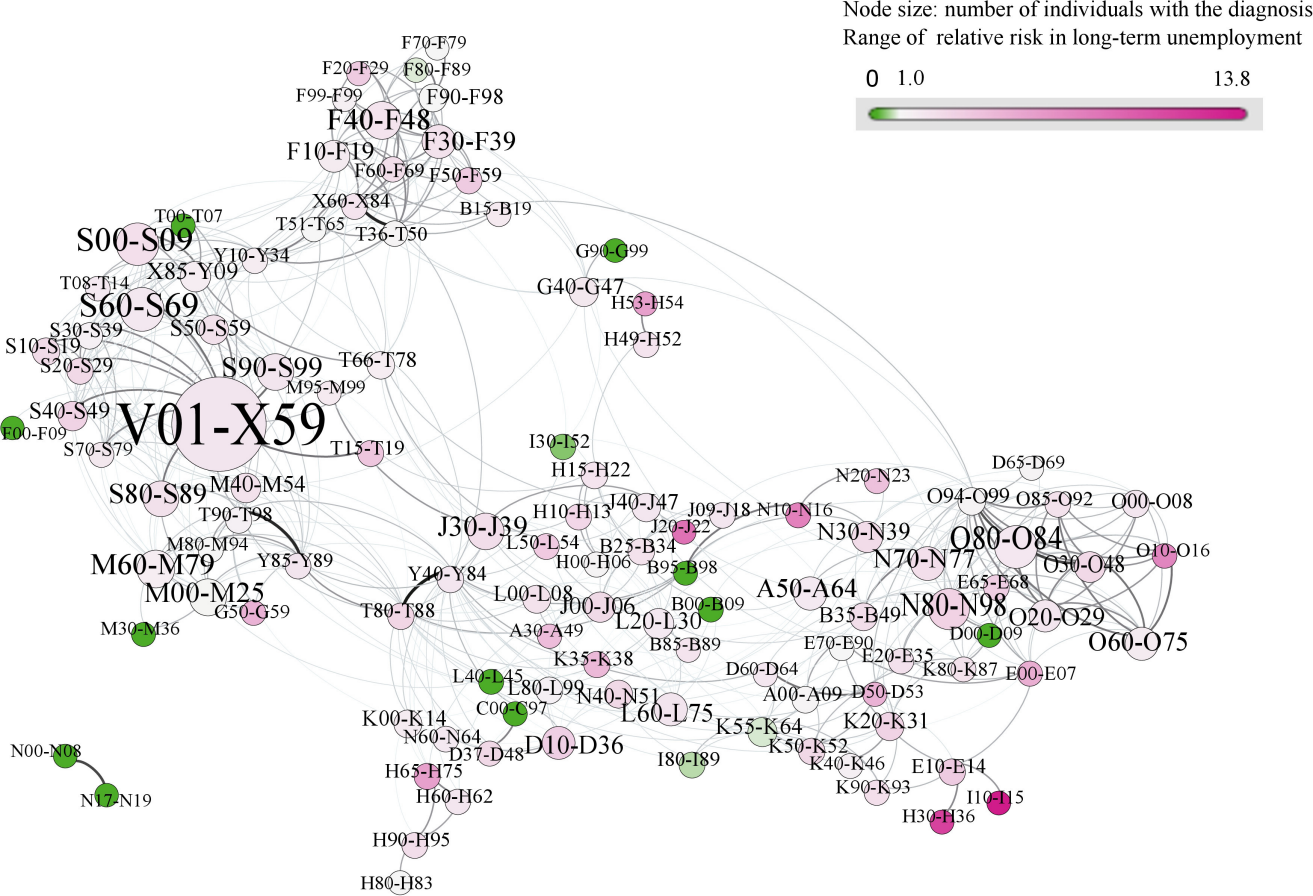
Multimorbidity network showing diagnose-specific relative risks for long-term unemployment in refugees vs. Swedish-born youths with common mental disorders (CMDs) (*n* = 249,245). In this multimorbidity network, each node (circle) encodes an ICD-10 diagnostic group, with a size given by the number of individuals with diagnoses in that diagnostic group, and colors giving the relative risk (RRs) for unemployment of refugee vs. the Swedish-born with common mental disorders (CMDs), respectively. Links between diagnostic groups indicate the tendency of co-occurrence in the same individual. The colors range from green to purple, corresponding to RRs from low to high. A node colored in green (purple) suggests that refugees with CMDs in that diagnostic group have a lower (higher) risk for long-term unemployment, compared to the Swedish-born counterparts. Overall, refugees with CMDs showed a higher risk for unemployment in the majority of diagnostic groups.

In addition, we identified the outcome difference between refugees and Swedish-born with CMDs in each diagnostic group by calculating the relative risk and 95% Confidence Intervals of LMM of young refugees with CMDs in each diagnostic group, compared to matched Swedish-born with CMDs. These relative risks were visualized by the color of nodes (diagnostic groups, ranging from green to purple) in the multimorbidity network (Figure 1). We further assessed the difference of relative risk between refugees and Swedish-born with CMDs using a Chi-squared test. Analyses of specific diagnoses with elevated risk of subsequent DP were not carried out due to the low number of cases among refugees with DP. All analyses were performed using R 3.6.2 and SAS 9.4, and an alpha value of <0.05 was considered as statistically significant in the disparity filter.

## Results

Table 1 presents the baseline characteristics of 249,245 individuals with a mean age (SD) of 23 (1.7) years, according to refugee status and CMDs. There were 20,680 (8.3%) diagnosed with CMDs. In the follow-up period 2,841 (1.1%) cases of DP and 16,323 (6.5%) cases of UE occurred. We observed a higher frequency of females than males among both the refugees and the Swedish-born with CMDs. We found a higher percentage of low educational level among the refugees than the Swedish-born, both for the refugees and the Swedish-born with CMDs (36.0 vs. 23.8%, respectively, *p* < 0.001), as well as for the refugees and the Swedish-born without CMDs (21.0 vs. 7.3%, respectively, *p* < 0.001). These findings were consistent with previous studies using the similar cohort (4, 7). Individuals with CMDs showed a higher proportion of previous sickness absences and previous UE compared with individuals without CMDs, respectively. Swedish-born individuals with CMDs showed the highest percentage of previous sickness absence (8.8%). Refugees with CMDs had the highest percentage of previous UE (12.2%) among the four groups.

**Table 1:** Baseline characteristics of individuals aged 20–25 years Sweden residents in 2011 (*n* = 249,245).

	**Refugees (*****n*** = **41,516)**		**Swedish-born**^**1**^ **(*****n*** = **207,729)**	
	**CMDs (*****n*** = **2,712)**	**No CMDs (*****n*** = **38,804)**	**CMDs** ***n*** = **17,968**	**No CMDs** ***n*** = **189,761**
Age (mean, sd)	22.8 (1.7)	22.6 (1.7)	22.8 (1.7)	22.6 (1.7)
Gender [male, *n* (%)]	1,177 (43.4)	21,012 (54.1)	6,630 (36.9)	104,352 (55.0)
**Family situation**, ***n*** **(%)**				
Married or cohabitant without children	133 (4.9)	1,994 (5.1)	221 (1.2)	1,846 (1.0)
Married or cohabitant with children	298 (11.0)	3,755 (9.7)	1,074 (6.0)	9,347 (4.9)
Single without children^2^	1,982 (73.1)	28,148 (72.5)	14,644 (81.5)	155,628 (82.0)
Single with children	153 (5.6)	1,114 (2.9)	564 (3.1)	1,902 (1.0)
Youth ( ≤ 20 years) living at home	146 (5.4)	3,793 (9.8)	1,465 (8.2)	21,038 (11.1)
**Area of residence**, ***n*** **(%)**				
Big cities	1,201 (44.3)	17,262 (44.5)	8,232 (45.8)	84,420 (44.5)
Medium-sized cities	1,084 (40.0)	15,918 (41.0)	7,286 (40.6)	77,749 (41.0)
Rural areas	427 (15.7)	5,624 (14.5)	2,450 (13.6)	27,592 (14.5)
**Education levels**, ***n*** **(%)**				
0–9 years	975 (36.0)	9,232 (23.8)	3,773 (21.0)	13,860 (7.3)
10–12 years	1,150 (42.4)	18,487 (47.6)	9,716 (54.1)	114,133 (60.2)
>12 years	469 (17.3)	9,415 (24.3)	4,417 (24.6)	61,296 (32.3)
Missing	118 (4.4)	1,670 (4.3)	62 (0.4)	472 (0.3)
Previous Sickness absences, *n* (%)^3^	135 (5.0)	332 (0.9)	1,573 (8.8)	1,953 (1.0)
Previous Unemployment, *n* (%)^4^	332 (12.2)	3251 (8.4)	924 (5.1)	3,933 (2.1)

1 Swedish-born indicates participants with both parents being native-born Swedes.

2 Single with/without children living at home.

3 Sickness absences was defined as whether an individual received spells of > 90 net days in 2009–2011.

4 Unemployment was defined as > 180 days unemployment in 2009–2011.

Table 2 shows the association of multimorbidity categories with the risk of subsequent DP among refugees with or without CMDs and Swedish-born with or without CMDs. The standardized median (range) multimorbidity scores were 0.20 (−0.48, 15.0) and 0.48 (−0.61, 17.7) in DP and UE, respectively. Relative to the reference group (Swedish-born without CMDs in the multimorbidity category “zero”), Swedish-born had higher odds of being granted DP than refugees across all CMDs and multimorbidity categories. CMDs and multimorbidity elevated the risk of DP considerably in Swedish-born with the highest OR [95%CI] being 13.8 [12.32, 15.59]. We were unable to estimate the OR of DP among refugees with CMDs due to low case numbers in the multimorbidity categories “zero” and “low.” The odds of UE remained higher in refugees across all three multimorbidity categories, compared to the Swedish-born. CMDs showed a tendency toward stronger risk than high multimorbidity for UE in Swedish-born, (OR [95% CI] 2.10 [1.95, 2.26] vs. 1.87 [1.63, 2.15], “high” vs. “zero” multimorbidity) whereas multimorbidity showed higher risk than CMDs toward UE in refugees. The pseudo-R^2^ values of the underlying multivariate regression models for DP and UE were 0.18 and 0.12, respectively.

**Table 2:** Odds ratios (95% Confidence Intervals) regarding subsequent disability pension and long-term unemployment (2012–2016) according to three multimorbidity categories, stratified by refugee status and common mental disorders (CMDs) in 249,245 young adults in Sweden.

		**Multimorbidity category zero (*****n*** = **124,590)**			**Multimorbidity category low (*****n*** = **62,323)**			**Multimorbidity category high (*****n*** = **62,323)**		
**Outcome**	**CMDs and refugee status**	**Case/n**	**Crude**	**Multivariate** ^1^	**Case/n**	**Crude**	**Multivariate**	**Case/n**	**Crude**	**Multivariate**
Disability pension (*n* = 2,841)	No CMDs Swedish-born	443/101,545	1	1	300/48,737	1.41 [1.22, 1.64]	1.38 [1.19, 1.59]	422/39,479	2.47 [2.16, 2.82]	1.96 [1.71, 2.25]
	CMDs Swedish-born	101/3,095	7.70 [6.15, 9.54]	6.27 [5.00, 7.79]	94/2,669	8.33 [6.61, 10.39]	6.87 [5.44, 8.60]	1102/12204	22.7 [20.3, 25.4]	13.8 [12.32, 15.59]
	No CMDs Refugee	72/19,605	0.84 [0.65, 1.07]	0.47 [0.36, 0.60]	75/10,568	1.63 [1.27, 2.07]	0.89 [0.69, 1.13]	83/8,631	2.22 [1.74, 2.79]	1.02 [0.79, 1.29]
	CMDs Refugee	< 10/345	4.04 [1.59, 8.32]	*^2^	< 10/349	5.35 [2.42, 10.14]	*	135/2,018	16.36 [13.38, 19.89]	6.91 [5.60, 8.48]
		**Multimorbidity category zero (*****n*** = **124,590)**			**Multimorbidity category low (*****n*** = **63,560)**			**Multimorbidity category high (*****n*** = **61,095)**		
**Outcome**	**CMDs and refugee status**	**Case/n**	**Crude**	**Multivariate** ^1^	**Case/n**	**Crude**	**Multivariate**	**Case/n**	**Crude**	**Multivariate**
Long-term unemployment (*n* =16,323)	No CMDs Swedish-born	3,809/101,545	1	1	1,792/48,135	0.99 [0.94, 1.05]	0.97 [0.92, 1.03]	1,896/40,081	1.27 [1.20, 1.35]	1.10 [1.04, 1.16]
	CMDs Swedish-born	240/3,095	2.16 [1.88, 2.46]	1.87 [1.63, 2.15]	356/4710	2.10 [1.87, 2.35]	1.75 [1.56, 1.96]	1,089/10,163	3.08 [2.87, 3.30]	2.10 [1.95, 2.26]
	No CMDs Refugee	2,862/19,605	4.39 [4.17, 4.62]	3.21 [3.04, 3.39]	1,674/10,038	5.13 [4.82, 5.46]	3.70 [3.47, 3.95]	2,004/9,161	7.18 [6.77, 7.62]	4.51 [4.23, 4.80]
	CMDs Refugee	64/345	5.84 [4.41, 7.63]	3.78 [2.81, 5.00]	120/677	5.53 [4.51, 6.72]	3.31 [2.67, 4.07]	417/1690	8.41 [7.48, 9.42]	4.75 [4.20, 5.35]

1 Multivariable model adjusted for education, sickness absence during 2009–2011, long-term unemployment during 2009–2011.

2 Low number of cases to carry out the multivariable model.

Potential two- and three-way interactions between CMDs, multimorbidity and refugee status were assessed. CMDs and multimorbidity significantly interacted (*p* = 0.0049) in DP. Regarding UE, the interactions between CMDs and refugee status, as well as between refugee status and multimorbidity were significant (*p* < 0.0001). Yet, the three-way interaction of multimorbidity, CMD, and refugee status was neither significant for DP (*p* = 0.52) nor for UE (*p* = 0.45).

Table 3 presents the most common diagnostic groups among refugees and Swedish-born individuals with CMDs and without CMDs. Accidents and external injuries of various parts of the body (ICD-10 Chapter S) were the most frequently occurring diagnostic groups in refugees and Swedish-born young adults. In individuals with CMDs, diagnostic groups containing CMDs such as neurotic, stress-related, and somatoform disorders (F40–F48) and mood [affective] disorders (F30–F39) were the most frequently occurring diagnostic groups in refugees and Swedish-born young adults, next to the no CMDs diagnostic group of accidents. Diagnostic groups that showed differences between refugee status and CMDs were mostly pregnancy and female-sex-related diagnoses (Chapter O, N80–N98, N70–N77). Overall, there is a clear tendency toward higher frequencies in the CMD group across all diagnostic groups in refugees and Swedish-born, suggesting a link between CMD and increased multimorbidity in these cohorts.

**Table 3:** The top 20 most frequent ICD-10 diagnostic groups (*n* = 249,245).

		**Refugees (*****n*** = **41,516)**		**Swedish-born**^**1**^ **(*****n*** = **207,729)**		***p*-value**
**ICD-10 disease groups**	**Description**	**CMDs** ^3^	**No CMDs**	**CMDs**	**No CMDs**	
V01-X59	Accidents	571 (21.1)	5650 (14.6)	3706 (20.6)	31017 (16.3)	0.0004
S60-S69	Injuries to the wrist and hand	179 (6.6)	1618 (4.2)	1120 (6.2)	8846 (4.7)	0.1214
O80-O84	Delivery	268 (9.9)	2986 (7.7)	1151 (6.4)	6770 (3.6)	0.0000
S00-S09	Injuries to the head	231 (8.5)	1581 (4.1)	1153 (6.4)	8077 (4.3)	0.7928
N80-N98	Noninflammatory disorders of female genital tract	273 (10.1)	1694 (4.4)	1639 (9.1)	6568 (3.5)	0.0000
F40-F48^2^	Neurotic, stress-related and somatoform disorders	1342 (49.5)	33 (0.1)	6832 (38.0)	116 (0.1)	0.0792
M60-M79	Soft tissue disorders	149 (5.5)	1141 (2.9)	978 (5.4)	6027 (3.2)	0.0227
M00-M25	Arthropathies	83 (3.1)	955 (2.5)	661 (3.7)	6120 (3.2)	0.0829
S90-S99	Injuries to the ankle and foot	77 (2.8)	1021 (2.6)	680 (3.8)	5874 (3.1)	0.0007
J30-J39	Other disease of upper respiratory tract	120 (4.4)	1197 (3.1)	748 (4.2)	5513 (2.9)	0.0039
S80-S89	Injuries to the knee and lower leg	81 (3.0)	979 (2.5)	570 (3.2)	5529 (2.9)	0.0848
N70-N77	Inflammatory diseases of female pelvic organs	159 (5.9)	890 (2.3)	1003 (5.6)	3957 (2.1)	0.0002
F30-F39^2^	Mood [affective] disorders	720 (26.5)	17 (0.04)	5048 (28.1)	168 (0.1)	0.2204
A50-A64	Infections with a predominantly sexual model of transmission	82 (3.0)	610 (1.6)	642 (3.6)	4388 (2.3)	0.5373
O60-O75	Complications of labor and delivery	144 (5.3)	1547 (4.0)	582 (3.2)	3378 (1.8)	0.0000
L60-L75	Disorders of skin appendages	119 (4.4)	1166 (3.0)	560 (3.1)	3749 (2.0)	0.0004
D10-D36	Benign neoplasms	59 (2.2)	632 (1.6)	523 (2.9)	4289 (2.3)	0.0724
O20-O29	Other maternal disorders predominantly related to pregnancy	196 (7.2)	1521 (3.9)	731 (4.1)	2593 (1.4)	0.0000
F10-F19	Mental and behavioral disorders due to psychoactive substance uses	233 (8.6)	346 (0.9)	2030 (11.3)	2257 (1.2)	0.0015
N30-N39	Other diseases of urinary system	121 (4.5)	719 (1.9)	603 (3.4)	2959 (1.6)	0.0848
O30-O48	Maternal care related to the fetus and amniotic cavity and possible delivery problems	131 (4.8)	1250 (3.2)	503 (2.8)	2355 (1.2)	0.0000

1 Swedish-born indicates individuals with both parents being native Swedes.

2 F30–F39, F40–F48 includes the ICD-10 codes of common mental disorders.

3 CMDs indicates common mental disorders.

Figure 1 shows the network of interrelations of diagnostic groups, together with their frequencies and relative risks for UE. The network reveals the existence of several densely connected (i.e., frequently co-occurring) diagnostic groups, referred to as diagnosis clusters. Diagnoses from a cluster tend to co-occur in individual patients, whereas there is substantially less tendency for two diagnoses belonging to different clusters to co-occur in a patient. The size of the node indicates the number of individuals that had a diagnosis within the given diagnostic group; the color of the node indicates the relative risk of UE among refugees with CMDs, compared to Swedish-born with CMDs. Diagnostic groups related to mental disorders (Chapter F, including CMDs) formed a densely connected diagnosis cluster; one diagnosis cluster consisted of poisonings and external causes of morbidity (Chapters S, T, and V–X); whereas another cluster of diagnostic groups emerged around pregnancy-related diagnoses (Chapter O), partially overlapping the cluster of disease of the genitourinary system (Chapter N).

The ICD-10 codes for all diagnostic groups in the network are listed in Supplementary Table 1. The dominant purple color in the multimorbidity network (Figure 1) shows that refugees with CMDs had a higher relative risk of UE, compared to Swedish-born with CMDs in the vast majority of diagnostic groups. Amongst all diagnostic groups, two diagnostic groups demonstrated particularly high relative risk of UE. Schizophrenia, schizotypal and delusional disorders (F20–F29) had 3.46 times (CI [1.77, 6.75]) higher risk of UE in refugees with CMDs as compared to the Swedish-born with CMDs. Refugees with CMDs who had behavioral syndromes associated with physiological disturbances and physical factors (F50–F59) showed 3.41 times (CI [1.90, 6.10]) the risk of UE compared to their counterparts (Supplementary Table 2).

## Discussion

### Main findings

This registry-based matched cohort study of 249,245 refugee and Swedish-born young adults leveraging a disease network approach, had two main aims. The first aim was to evaluate the association of multimorbidity, CMD, and refugee status with subsequent LMM, whereas the second aim was to identify diagnostic groups with an elevated risk of LMM in refugee and Swedish-born young adults with CMDs. As for the first aim, we found that multimorbidity was associated with LMM. The findings suggest that refugees had lower odds of DP and higher odds of UE than Swedish-born. While there was a strong association between multimorbidity score and CMDs with DP among Swedish-born, CMDs but not multimorbidity showed elevated risk toward UE in Swedish-born. Multimorbidity in the presence of CMDs suggested a stronger influence on subsequent UE in refugees. There was a significant interaction between CMDs and multimorbidity in DP (*p* = 0.0049), whereas CMDs and refugee status, as well as refugee status and multimorbidity significantly interacted in UE (*p* < 0.0001). As for the second aim, the majority of diagnostic groups showed higher relative risks of UE for young refugees with CMDs, compared to their Swedish-born counterparts.

We found a lower risk of DP and higher risk of UE in refugees than Swedish-born young adults across all CMDs and multimorbidity categories. These findings are consistent with a previous study examining the relation of multimorbidity with LMM among refugee youths and Swedish-born, and might be attributable to several mediators (24). First, lower socioeconomic status was associated with less granted DP (5, 32). Limited social support and inadequate knowledge of the social insurance system might potentially place refugees at a disadvantage for effectively seeking DP (33). Second, parental support plays a role in receiving DP in early adulthood (23, 34, 35). Previous studies suggested that young adults with higher-educated parents have better family support and access to care (34, 36). Disparities in health education of parents may lead to disparities in early identification of child or adolescent care needs, which could then contribute to disparities in DP during early adulthood. Further, the high barrier to successfully receiving DP may lead to increased utilization of other forms of social security support among socially disadvantaged groups. These disparities in access to DP suggest that the number of refugees who are in need of DP might be underestimated. Lastly, young refugees were more likely to have lower educational attainment, language barriers, social stigma, and marginalization (8). These hardships make it more challenging to obtain a job (8), and may contribute to the higher UE rate in refugee youths at baseline regardless of CMD diagnosis. Nevertheless, another Swedish cohort study showed a higher risk of DP in refugees with specific diagnoses than the native-born in all ages (6). This discrepancy may be due to methodological design, the adjustment of confounders in the models, and potential effect modification by age due to our study focus specifically in young adults. While the previous study examined the specific mental and somatic disorders with DP, our study accounted for all the co-occurring diagnoses by conceptualizing into a multimorbidity score.

The present study suggests that co-occurrence of CMDs with other coexisting mental or somatic diagnoses (referred to as multimorbidity) elevated the risk of subsequent DP in young adults, particularly in Swedish-born youths. Diseases may coexist due to another, confounding condition, random chance, or selection bias: random chance refers to etiologically unrelated diagnoses that develop independently; selection bias refers to the greater likelihood of identifying a new diagnosis among individuals seeking medical care (18, 37). This increased overall disease burden within an individual could impede the engagement in the labor market (24, 38, 39). However, potential causal relations between CMDs and other chronic conditions with regard to work productivity loss have not yet been fully understood. Anxiety and mood disorders were found to have an additive effect with most physical disorders in terms of functional impairment and work loss in a previous study (38), whereas other studies suggested a synergistic effect on work productivity loss and DP (22, 28). Moreover, perceived health status, high job demands, and stress may prolong the duration of sick leave among individuals with CMDs (40). Furthermore, granting DP requires medical assessments from specialists to confirm reduced work capacity (41), meaning that this outcome variable can be expected to be more closely related to higher medical severity than UE. Due to the large number of affected youths and impact upon the labor market integration, promotion of awareness of comorbid mental and somatic disorders with CMDs, comprehensive assessments, and enhanced collaboration between psychiatric and somatic health care are crucial for preventing DP.

In contrast to our result for DP but in line with a previous study investigating the relation of refugee status and specific mental disorders with LMM, CMD status added no further risk to UE in refugee youths, while Swedish-born youth had an increased risk of UE with diagnosis of CMD (7). This study adds that multimorbidity, in addition to CMD, does not have an influence on the subsequent risk of UE in either refugees or Swedish-born. UE is further associated with other residual confounding measures, such as discrimination and language proficiency, putting refugee youths at a disadvantage in the labor market (8). Refugee or migration status alone were shown to be strong risk factors for UE, with estimated two to three times higher likelihood of UE than for their native-born counterparts without CMDs (5, 7). The greater severity of mental disorders due to the migration experience may result in obtaining disability benefits other than UE (42).

The multimorbidity network revealed higher relative risk of UE in refugees with CMDs compared to Swedish-born in most diagnostic groups. In particular, delusional and behavior syndromes related disorders showed the highest relative risk increase. Lack of adequate support from the health care system and labor market may contribute to this observation. Moreover, somatic disorders might be underdiagnosed due to inadequate treatment of CMDs among the refugee young adults, which may further marginalize this vulnerable group (36, 43, 44).

### Methodological discussion

Strengths of the current study include the large sample size, long follow-up time, nationwide high quality of validated register data (45), prospective design, as well as the novel network approach in computing the multimorbidity score. Our study also has limitations. First, we lacked statistical power to study DP with CMDs across various multimorbidity categories for refugee young adults. Second, diagnostic data was constrained to inpatient and specialized outpatient care. Young refugees showed lower healthcare utilization compared to their counterparts in most mental disorders (46). The included refugees of this study might therefore experience higher severity of their diseases. Treatment of affective disorders often occurs in the primary care setting. Thus, the use of antidepressants might have been one of the criteria of CMD to account for the lack of information in primary health care. Third, data describing the disease severity was not collected in our study. Moreover, our study focuses on refugee and Swedish-born young adults in Sweden and labor market welfare support, which may not be generalizable to countries with different social insurance systems. Furthermore, CMDs among refugees may not be detected to the same extent as in their Swedish counterparts due to educational differences and a lack of consideration of cultural diversity competences in the assessment by health care professionals and other barriers such as stigmatization. Perceived CMDs symptoms differ by cultural background and could be underdiagnosed given a lack of culturally sensitive instruments (43, 44). This may lead to lower psychiatric utilization and inadequate screening among refugee youths. Providing transcultural medicine training for healthcare professionals could decrease the bias in clinical settings and increase access to healthcare utilization for refugees. Hence, promoting accessible intervention to address CMDs in refugee youths and improving awareness of cultural factors in the medical assessment are essential.

## Conclusion

The co-occurrence of CMDs and multimorbidity was strongly associated with higher risk of DP in Swedish-born young adults. The majority of diagnostic groups in the multimorbidity network suggested an elevated risk of UE in young refugees with CMDs compared to their counterparts. These findings elucidate how multimorbidity in individuals with or without CMDs contribute to LMM and offer leverage points for targeted intervention and supportive prevention from primary and specialist care providers.

## Data availability statement

The data analyzed in this study is subject to the following licenses/restrictions: The highly sensitive microdata used in this study cannot be made publicly available, according to several laws, such as the General Data Protection Regulation, the Swedish law SFS 2018:218, the Swedish Data Protection Act, the Swedish Ethical Review Act, and the Public Access to Information and Secrecy Act. Requests to access these datasets should be directed to Karolinska Institutet, the Division of Insurance Medicine through Prof Kristina Alexanderson [PI of the database (contact via Kristina.alexanderson@ki.se)].

## Ethics statement

The studies involving human participants were reviewed and approved by the study was conducted in accordance with the World Medical Association Declaration of Helsinki. Participant consent is generally not required in large register-based studies in the Nordic countries and was waived by the Regional Ethical Review Board in Stockholm, Sweden, who approved of the project (approval numbers: 2007/762-31; 2009/23-32, 2009/1917-32; 2010/466-32; 2011/1710-32; 2011/806-32; 2016/1533-32). All data were anonymized by the administrative authorities before delivered to us. Written informed consent for participation was not required for this study in accordance with the national legislation and the institutional requirements.

## Author contributions

PK, EM-R, and JC contributed to the concept, methods, and results interpretation of the study. JC was responsible for data analysis and drafting the manuscript. All authors contributed to constructive comments and critical revision of the manuscript. All authors approved the final version.
