# Supplementary material for: Association of common mental disorders and related multimorbidity with subsequent labor market marginalization among refugee and Swedish-born young adults
Source: Front Public Health. 2023 Mar 16;11:1054261. doi: 10.3389/fpubh.2023.1054261 (PMC10060531; doi:10.3389/fpubh.2023.1054261)
Supplement: Supplementary file 1 [file Table_1.DOCX]

**Supplementary Table 1** List of ICD-10 diagnostic groups in the multimorbidity network

| ICD-10 diagnostic groups^1^ | Diagnoses Name |
| --- | --- |
| A00-A09 | Intestinal infectious disease |
| A30-A49 | Other bacterial diseases |
| A50-A64 | Infections with a predominantly sexual model of transmission |
| B00-B09 | Viral infections characterized by skin and mucous membrane |
| B15-B19 | Viral hepatitis |
| B25-B34 | Other viral diseases |
| B35-B49 | Mycoses |
| B85-B89 | Pediculosis, acariasis and other infestations |
| B95-B98 | Bacterial, viral and other infectious agents |
| C00-C97 | Malignant neoplasms |
| D00-D09 | In situ neoplasms |
| D10-D36 | Benign neoplasms |
| D37-D48 | Neoplasms of uncertain or unknown behavior |
| D50-D53 | Nutritional anemias |
| D60-D64 | Aplastic and other anemias |
| D65-D69 | Coagulation defects, purpura and other hemorrhagic condition |
| E00-E07 | Disorder of thyroid gland |
| E10-E14 | Diabetes mellitus |
| E20-E35 | Disorders of other endocrine glands |
| E65-E68 | Obesity and other hyperalimentation |
| E70-E90 | Metabolic disorders |
| F00-F09 | Organic, including symptomatic, mental disorders |
| F10-F19 | Mental and behavioral disorders due to psychoactive substance uses |
| F20-F29 | Schizophrenia, schizotypal and delusional disorders |
| F30-F39 | Mood [affective] disorders |
| F40-F48 | Neurotic, stress-related and somatoform disorders |
| F50-F59 | Behavioral syndromes associated with physiological disturbances and physical factors |
| F60-F69 | Disorders of adult personality and behavior |
| F70-F79 | Mental retardation |
| F80-F89 | Disorders of psychological development |
| F90-F98 | Behavioral and emotional disorders with onset usually occurring in childhood and adolescence |
| F99-F99 | Unspecified mental disorder |
| G40-G47 | Episodic and paroxysmal disorders |
| G50-G59 | Nerve, nerve root and plexus disorders |
| G90-G99 | Other disorders of the nervous system |
| H00-H06 | Disorders of eyelid, lacrimal system and orbit |
| H10-H13 | Disorders of conjunctiva |
| H15-H22 | Disorders of sclera, cornea, iris and ciliary body |
| H30-H36 | Disorders of choroid and retina |
| H49-H52 | Disorders of ocular muscles, binocular movement, accommodation and refraction |
| H53-H54 | Visual disturbances and blindness |
| H60-H62 | Diseases of external ear |
| H65-H75 | Diseases of middle ear and mastoid |
| H80-H83 | Diseases of inner ear |
| H90-H95 | Other disorders of ear |
| I10-I15 | Hypertensive diseases |
| I30-I52 | Other forms of heart disease |
| I80-I89 | Diseases of veins, lymphatic vessels and lymph nodes, not elsewhere classified |
| J00-J06 | Acute upper respiratory infections |
| J09-J18 | Influenza and pneumonia |
| J20-J22 | Other acute lower respiratory infections |
| J30-J39 | Other disease of upper respiratory tract |
| J40-J47 | Chronic lower respiratory disease |
| K00-K14 | Diseases of oral cavity, salivary glands and jaws |
| K20-K31 | Diseases of esophagus, stomach and duodenum |
| K35-K38 | Diseases of appendix |
| K40-K46 | Hernia |
| K50-K52 | Noninfective enteritis and colitis |
| K55-K64 | Other diseases of intestines |
| K80-K87 | Disorders of gallbladder, biliary tract and pancreas |
| K90-K93 | Other diseases of the digestive system |
| L00-L08 | Infections of the skin and subcutaneous tissue |
| L20-L30 | Dermatitis and eczema |
| L40-L45 | Papulosquamous disorders |
| L50-L54 | Urticaria and erythema |
| L60-L75 | Disorders of skin appendages |
| L80-L99 | Other disorders of the skin and subcutaneous tissue |
| M00-M25 | Arthropathies |
| M30-M36 | Systemic connective tissue disorders |
| M40-M54 | Dorsalgia |
| M60-M79 | Soft tissue disorders |
| M80-M94 | Osteopathies and chondropathies |
| M95-M99 | Other disorders of the musculoskeletal system and connective tissue |
| N00-N08 | Glomerular diseases |
| N10-N16 | Renal tubulo-interstitial diseases |
| N17-N19 | Renal failure |
| N20-N23 | Urolithiasis |
| N30-N39 | Other diseases of urinary system |
| N40-N51 | Diseases of male genital organs |
| N60-N64 | Disorders of breast |
| N70-N77 | Inflammatory diseases of female pelvic organs |
| N80-N98 | Noninflammatory disorders of female genital tract |
| O00-O08 | Pregnancy with abortive outcome |
| O10-O16 | Oedema, proteinuria and hypertensive disorders in pregnancy, childbirth and the puerperium |
| O20-O29 | Other maternal disorders predominantly related to pregnancy |
| O30-O48 | Maternal care related to the fetus and amniotic cavity and possible delivery problems |
| O60-O75 | Complications of labor and delivery |
| O80-O84 | Delivery |
| O85-O92 | Complications predominantly related to the puerperium |
| O94-O99 | Other obstetric conditions, not elsewhere classified |
| S00-S09 | Injuries to the head |
| S10-S19 | Injuries to the neck |
| S20-S29 | Injuries to the thorax |
| S30-S39 | Injuries to the abdomen, lower back, lumbar spine and pelvis |
| S40-S49 | Injuries to the shoulder and upper arm |
| S50-S59 | Injuries to the elbow and forearm |
| S60-S69 | Injuries to the wrist and hand |
| S70-S79 | Injuries to the hip and thigh |
| S80-S89 | Injuries to the knee and lower leg |
| S90-S99 | Injuries to the ankle and foot |
| T00-T07 | Injuries involving multiple body regions |
| T08-T14 | Injuries to unspecified part of trunk, limb or body region |
| T15-T19 | Effects of foreign body entering through natural orifice |
| T36-T50 | Poisoning by drugs, medicaments and biological substances |
| T51-T65 | Toxic effects of substances chiefly nonmedicinal as to source |
| T66-T78 | Other and unspecified effects of external causes |
| T80-T88 | Complications of surgical and medical care, not elsewhere classified |
| T90-T98 | Sequelae of injuries, of poisoning and of other consequences of external causes |
| V01-X59 | Accidents |
| X60-X84 | Intentional self-harm |
| X85-Y09 | Assault |
| Y10-Y34 | Event of undetermined intent |
| Y40-Y84 | Complications of medical and surgical care |
| Y85-Y89 | Sequalae of external causes of morbidity and mortality |
| ^1^ICD-10 diagnostic group indicates the International Classification of Diseases Tenth Edition provided by World Health Organization. | |

| **Supplementary Table 2. Diagnostic groups with the highest relative risk of unemployment in refugees with common mental disorders (CMDs) vs Swedish-born with common mental disorders** | | | | | | | | |
| --- | --- | --- | --- | --- | --- | --- | --- | --- |
| **Diagnostic Groups^1^** | Description | Refugee | Swedish-born | N | CMDs^2^ | Unemployment in refugee with CMDs | Unemployment in Swedish-born with CMDs | Relative Risk^3^ |
| **F20-F29** | Schizophrenia, schizotypal and delusional disorders | 156 | 299 | 455 | 301 | 18 (21%) | 13 (6%) | 3.46 [1.77,6.75] |
| **F50-F59** | Behavioral syndromes associated with physiological disturbances and physical factors | 178 | 1574 | 1752 | 1153 | 13 (14%) | 43 (4%) | 3.41 [1.90, 6.10] |
| **N80-N98** | Noninflammatory disorders of female genital tract | 1967 | 8207 | 10174 | 1912 | 37 (14%) | 73 (4%) | 3.04 [2.09, 4.42] |
| **K20-K31** | Diseases of oesophagus, stomach and duodenum | 831 | 2044 | 2875 | 587 | 22 (17%) | 27 (6%) | 2.95 [1.74, 5.00] |
| **O30-O48** | Maternal care related to the fetus and amniotic cavity and possible delivery problems | 1381 | 2858 | 4239 | 634 | 15 (11%) | 22 (4%) | 2.62 [1.40, 4.90] |
| **S20-S29** | Injuries to the thorax | 338 | 1389 | 1727 | 275 | 12 (20%) | 17 (8%) | 2.58 [1.31, 5.10] |
| **N30-N39** | Other diseases of urinary system | 840 | 3562 | 4402 | 724 | 13 (11%) | 27 (4%) | 2.40 [1.28, 4.53] |
| **F60-F69** | Disorders of adult personality and behavior | 128 | 1048 | 1176 | 1001 | 14 (14%) | 53 (6%) | 2.35 [1.36,4.09] |
| **S00-S09** | Injuries to the head | 1812 | 9230 | 11042 | 1384 | 37 (16%) | 81 (7%) | 2.28 [1.59, 3.28] |
| **N70-N77** | Inflammatory diseases of female pelvic organs | 1049 | 4960 | 6009 | 1162 | 20 (13%) | 56 (6%) | 2.25 [1.39, 3.65] |
| ^1^Diagnostic groups were filter by unemployment in refugee with common mental disorder greater than 10  ^2^CMDs: Common mental disorders  ^3^Relative risk: unemployment in refugees with CMDs vs. unemployment in Swedish-born with CMDs | | | | | | | | |

**Supplementary Table 3**. Distribution of multimorbidity score (MS)

Categorized MS in disability pension

| MS score | Frequency |
| --- | --- |
| <= -0.48 | 124,590 |
| <= 0.2 (median) | 62,323 |
| >0.20 (maximum score 15.0) | 62,323 |

Categorized MS in unemployment

| MS score | Frequency |
| --- | --- |
| <= - 0.61 | 124,590 |
| <=0.48 | 63,560 |
| >0.48 (maximum score 17.7) | 61,095 |

B

A

**Supplementary Figure 1**. An example of the computation of the multimorbidity score (MS) for an individual. Each circle indicates a diagnostic group, and the link weight, *w_ij_*, indicates the tendency of the co-occurrence of the diagnostic groups. This individual has two diagnoses, A and B, colored in red. Each node (diagnostic group) is further assigned a value that gives the LMM risk, *Y_j_*, associated with having any of these diagnoses measured at baseline. The MS of this individual is calculated as MS = $\sum w_{ij}Y_{j}$ where the sum ranges over all diagnoses i that the individual has at baseline. In this case, the MS would yield a result of 6.7.


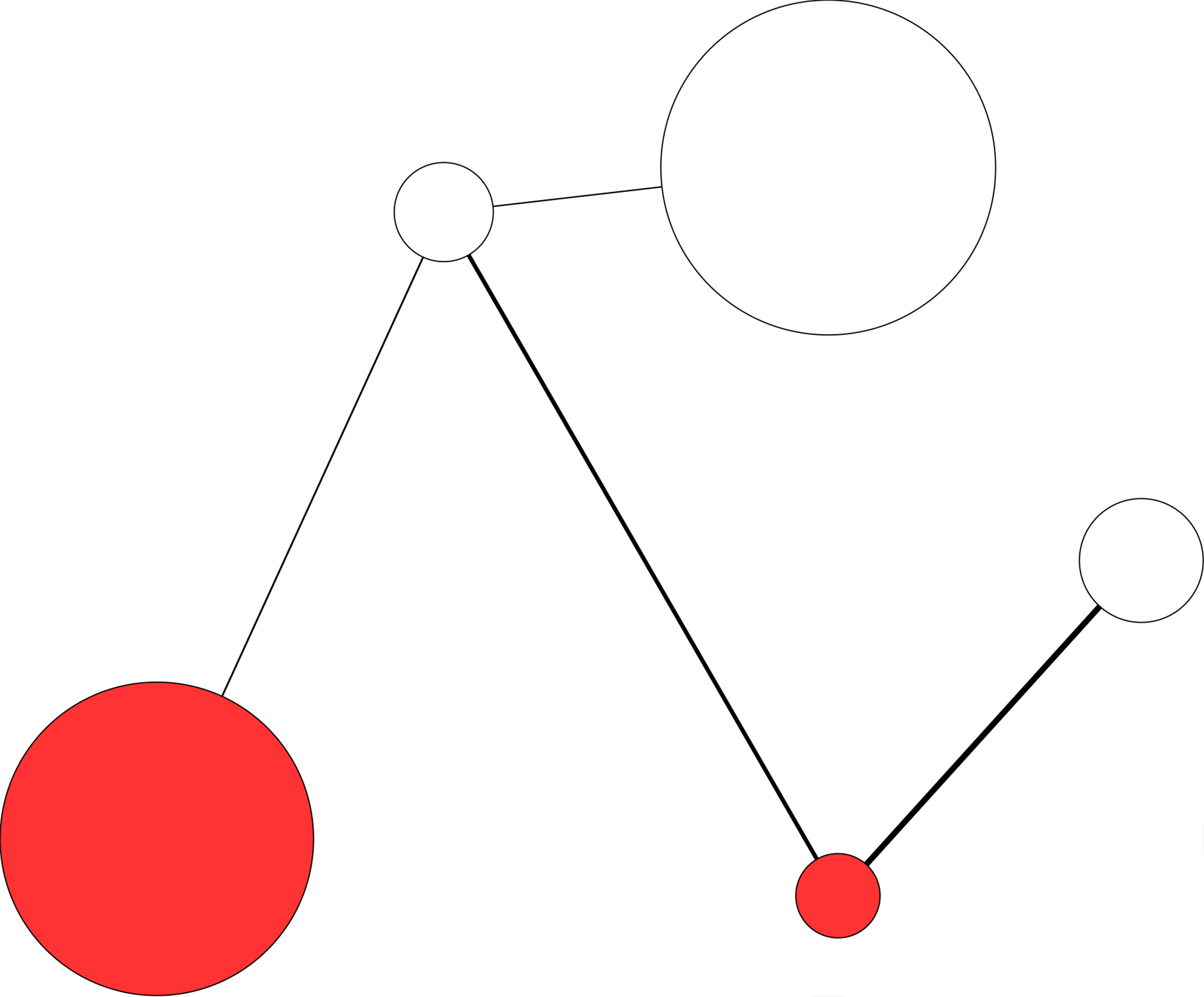


1.5

3.4

4.7

0.6

0.8

**Supplementary Figure 2.** Histogram of multimorbidity score (MS) for disability pension. We find a heavily right-skewed distribution in which most individuals have low multimorbidity score (as could be expected in our cohort of young adults) but few individuals have much higher scores.

MS in DP

**Supplementary Methods**

Regarding the construction of the disease networks, our method uses two thresholds to filter out rare diagnostic groups: the minimum number of patients with a given diagnostic group and the minimum number of cooccurrences that a diagnostic group has with any other group. Both numbers are cutoffs that aim to reduce spurious correlations from the disease network. Due to the systematic screening of all pair-wise disease associations, including rare diagnoses in the analysis would result in a large number of unreliable links with very high (or low) relative risks for which there are only a few observations. Filtering the network by keeping only links with a significant pair-wise disease association, on the other hand, would strongly bias the results toward the most frequent diagnoses. To balance these two tendencies (filtering by relative risk estimates favors rare diagnoses, p-values favor frequent diagnoses), we chose to remove rare diagnoses by cutoffs and used a comparison with diagnosis-specific null models (the disparity filter [Serrano MA, Boguna M, Vespignani A. Extracting the multiscale backbone of complex weighted networks. *Proceedings of the National Academy of Sciences*. 2009/04/08/ 2009:6483-6488 IS -16 VL -106. doi:10.1073/pnas.0808904106]) rather than pair-wise significance tests for each link to remove non-significant links from the network. The number of eligible pairs of diagnoses using different cut-offs was also examined.
